# Morphology and control roles in perturbed standing recovery: a robotic study

**DOI:** 10.3389/frobt.2026.1840011

**Published:** 2026-07-01

**Authors:** Yelin Jiang, Marc Murcia, Jitong Yang, Andre Seyfarth, Rolf Findeisen, Maziar A. Sharbafi

**Affiliations:** 1 CCPS, Department of Electrical Engineering and Information Technology, Technical University of Darmstadt, Darmstadt, Germany; 2 Lauflabor, Institute of Sports Science, Technical University of Darmstadt, Darmstadt, Germany

**Keywords:** biarticular muscles, bipedal robot, force feedback control, pneumatic artificial muscles, postural balance

## Abstract

Postural balance is essential for both humans and robots, as failures increase fall risk and limit robotic performance in real-world settings. Although humans and robots share fundamental balancing mechanics, biological complexity limits the isolation of individual muscle functions and direct principle transfer to robots. In this study, we use EPA-Walker, a bio-inspired robot actuated by electric motors and pneumatic artificial muscles (PAMs), as a physical platform to investigate perturbed standing. Here, we focus on the PAM-driven actuation to systematically examine the roles of muscle morphology and control, and to validate biomechanical findings in a robotic setting. To enable a clear upper body perturbation, a Control Moment Gyroscope (CMG) was integrated. We evaluated two stabilization paradigms: passive standing, in which joint compliance was tuned through static PAM pressurization, and active balancing, in which a bio-inspired ground reaction force (GRF) feedback controller generated muscle reflexes. The results showed that biarticular thigh muscles, particularly the hamstrings and rectus femoris, played the most prominent role in enhancing robustness through both morphology in the passive experiments and reflex control in the active experiments, consistent with findings from human perturbation studies. While ankle muscles, such as the soleus, were essential for stable standing mainly through their passive morphological contribution, their reflex-based action could also improve robustness in a more specific manner through center-of-pressure regulation. Activating a single muscle could significantly improve robustness beyond morphology, enabling recovery from 3 Nm perturbations. Furthermore, synergistic reflex of biarticular muscles, especially hamstrings and gastrocnemius, extends the robustness to larger perturbations (5 Nm) . Our contribution highlights the synchronization of control strategies with the underlying morphological design through a universal sensory feedback signal, namely, GRF. These findings demonstrate the value of bio-inspired robots as testbeds to understand the potential principles underlying human motor control and support the transfer of such principles to legged robots and assistive systems.

## 1 Introduction

Robust upper-body postural control is essential for bipedal locomotion across diverse terrains and complex scenarios (Peng et al., 2017; Joe and Oh, 2019; Radosavovic et al., 2024). Failure to maintain balance causes fall-related injuries, even mortality in humans Hausdorff et al. (2001), Rubenstein and Josephson (2002), and critically limits the scope of bipedal robot applications in real-world unknown environments (Castano et al., 2022; Dan et al., 2025). As a fundamental prerequisite for locomotion gaits, standing is inherently unstable and deserves particular attention, especially under external perturbations. To keep upright postures, biological systems leverage the interplay of intrinsic muscle compliance, reflex-mediated feedback, and coordinated biarticular and monoarticular muscle action (Mohseni et al., 2024; Mohseni et al., 2025)), offering principles that can inform robotic systems (Nejadfard et al., 2020; Sharbafi et al., 2016; Nejadfard et al. 2019). Conversely, robotic investigation may provide reciprocal insight into human motor control (Sivak et al., 2026; Murcia et al., 2025).

Perturbed standing typically involves two main aspects: targeted models and perturbation techniques. In biomechanics, quiet standing is often studied using reduced-order models, for example, the inverted pendulum (van der Kooij et al., 1999; Mergner et al., 2003; Welch and Ting, 2008; Sarmadi et al., 2017) and multi-link inverted pendulum models (Suzuki et al., 2012; Kot and Nawrocka, 2016). While computationally efficient, they fail to capture complex musculoskeletal morphology and neural-motor control. Conversely, detailed neuromusculoskeletal models (Zhu et al., (2023) incorporate musculoskeletal dynamics, yet are difficult to implement in real-world experiments or humanoid robots. To evaluate system robustness, external perturbations are commonly used Horak et al. (1997); Mergner (2010). In the real-world disturbances, such as push recovery Fujimoto et al. (2015); Vlutters et al. (2016) and support surface translation Mergner (2010); Welch and Ting (2008), previous studies have quantified the responses and analyzed the underlying neuromuscular reactions that contribute to postural stability. However, these conventional perturbations typically involve horizontal force components that displace the leg axes and induce cross-talk between multiple response mechanisms, thereby hindering the isolation of upper body rotation. Pure torque perturbations on the torso, which were investigated in human movements Schumacher et al. (2019); Mohseni et al. (2025), could provide clearer understandings into upper-body balancing.

Parallel to biomechanics, robot standing controllers can be designed via template-based models Noh et al. (2004); Pratt et al. (2006); Li et al. (2021) or full-dynamics models Herzog et al. (2014); Heng et al. (2025). These approaches allow bipeds to maintain balance through satisfying dynamic constraints, but their performances are heavily dependent on model accuracy and computational resources. In recent years, state-of-the-art robust controllers increasingly involve learning-based techniques (Bong et al. (2022); Li et al., 2025), Zhang et al. (2025), such model-free approaches, however, are black-box methods that lack physical interpretability. More importantly, most existing frameworks rely on rigid motor actuation, diverging significantly from biological compliance.

Despite the advances in modeling and control, a critical gap remains: investigating the specific interplay of muscle morphology and control under uncoupled, pure upper-body perturbations in the real world is still unexplored. Previous human experiments (Fujimoto et al., 2015; Schumacher et al., 2019) attempted to apply pure torque perturbations to humans standing, successfully highlighting the contributive reflex activities of thigh biarticular muscles. While this offered valuable physiological insights, human experiments inherently lack the ability to isolate and selectively disable specific muscles or pathways for functional investigation. Bio-inspired robot offers a promising approach to bridge this gap. By integrating morphological and control similarity, bio-inspired robots demonstrate both locomotion versatility and the capacity for reverse-engineering biological principles (Liu et al., 2018; Takahashi et al., 2023; Jiang et al., 2024). To realize this approach, we introduced the hybrid electric-pneumatic actuator (EPA) Ahmad Sharbafi et al. (2017) to develop bioinspired robots such as EPA-Walker (Silva et al., 2024). The design of this robot has extensively drawn inspiration from humans, featuring human-like skeletal structures, biomimetic mass distribution, and compliant pneumatic artificial muscles (PAMs).

In this paper, we bridge the aforementioned gap by systematically investigating muscle morphology and control functions during passive and active perturbed standing experiments using the EPA-Walker platform. we utilized the Control Moment Gyroscope (CMG) Marquardt et al. (2021) to generate pure torque perturbations, with the aim of isolating the underlying rotational balance mechanisms, focusing on fundamental principles rather than direct real-world scenario reproduction. Building upon the most robust passive configuration, we proposed a positive ground reaction force (GRF) feedback controller across different PAM groups (Sharbafi and Seyfarth, 2015). The inspiration and theoretical foundation come from the Virtual Pivot Point (VPP) concept that translates system dynamics representation from an unstable inverted pendulum to a regular virtual pendulum (Maus et al., 2010). The core contribution of our study is threefold: (1) Successful integration of a CMG onto a bio-inspired bipedal robot for physically decoupled upper-body balance testing, (2) Development and physical implementation of a novel horizontal-GRF-based reflex controller on a PAM-driven robot, and (3) Systematic investigation of muscle morphology and reflex pathways to decode the mechanisms of human standing balance.

## 2 Methods

### 2.1 EPA with CMG: Platform for perturbation investigation

**FIGURE 1:**
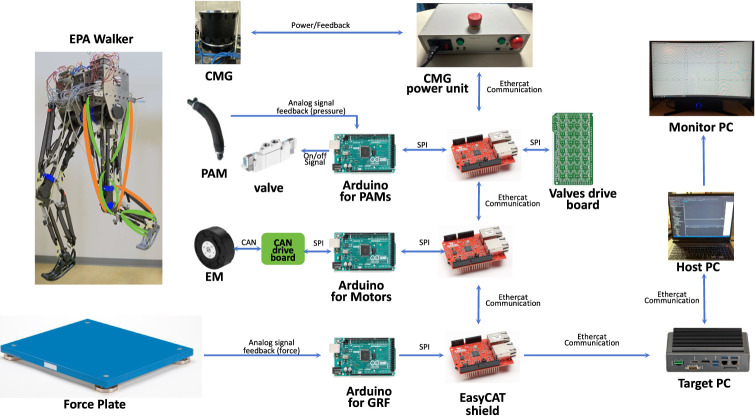
Overview of the EPA-Walker bipedal robot and the hardware architecture. Monoarticular muscles are highlighted in green, and biarticular muscles are highlighted in orange.

The EPA-Walker Silva et al. (2024) is a bioinspired bipedal robot that uses hybrid Electric Pneumatic Actuation (EPA), integrating electric motors and pneumatic artificial muscle (PAM) (Figure 1 left side). The utilization of parallel PAMs alongside rigid motors introduces intrinsic mechanical compliance, capable of replicating the biological adaptability and musculoskeletal intelligence found in human motor control (Ahmad Sharbafi et al., 2017).

In each leg, nine representative muscle groups are characterized based on their anatomical functions. As shown in Figure 1, six of them are monoarticular muscles (green), consisting of flexors (IL, POP, TIB) and extensors (GLU, VAS, SOL) that span a single joint. In addition, three biarticular muscles (HAM, RF, GAS; orange) are incorporated, each spanning two joints to provide inter-joint coordination. To ensure sufficient torque generation for static balance, two parallel SOL and TIB PAMs were included at the ankle joint. The internal chambers of these redundant pairs are interconnected to share valves and pressure sensors, ensuring synchronized force output. The EPA-Walker also integrates two electric motors at each leg (GIM8115-9), which are deactivated during the current study to isolate the functional contributions of the muscular system during balance recovery.

To implement controlled disturbances, a Control Moment Gyroscope (CMG) developed by TU Delft Meijneke et al. (2021) is integrated into the robot. The mass of the CMG is 1.2 kg with a maximum peak torque of 15 Nm . It is centrally located in the trunk to ensure symmetric transmission of angular momentum and generate pure torque perturbations for quantitative analysis. More details of the robot parameters and CMG are described in Supplementary Appendix 1.

The pneumatic system is regulated by on-off valves (SYJ3320, SMC) and monitored by pressure sensors (PSE530, SMC). GRF is measured using force plates (Type 9260AA, Kistler) and filtered at 5 Hz to provide feedback for reflex-based strategies. Kinematic data were acquired using a Qualisys motion capture system (not shown) at 100 Hz . Nine markers were placed, two on the trunk, and one on the hip, thigh, knee, shank, ankle, heel, and toe. All hardware modules, including sensors and valves, are integrated via EtherCAT for real-time synchronization. TwinCAT 4024 on a Host/Target PC is utilized for real-time control and data monitoring at a frequency of 1000 Hz , with control code developed and compiled in MATLAB 2023a.

### 2.2 GRF feedback for muscle reflex control

**FIGURE 2:**
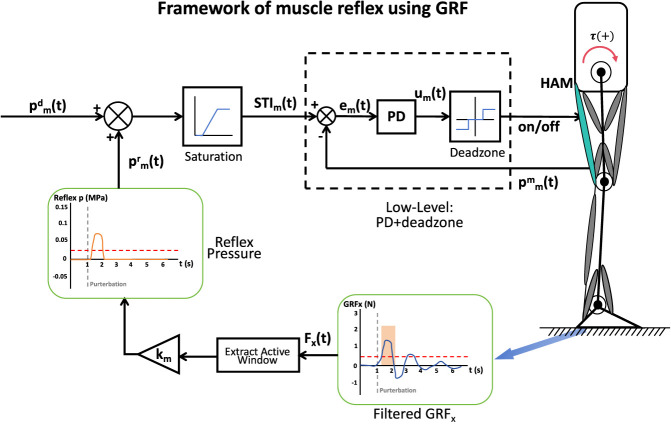
Framework of the active balancing strategy. The hierarchical control architecture consists of a high-level reflex loop using horizontal GRF and a low-level pressure regulator (HAM muscle is shown in cyan as an example).

We implemented a GRF positive feedback control strategy, as illustrated in Figure 2. The control law is inspired by the evidence of positive force feedback, a mechanism widely observed in stable bipedal locomotion (Prochazka et al., 1997; Geyer et al., 2003; Grey et al., 2007). In our initial approach, we utilized local muscle pressure as a feedback strategy. While doing the first experiments with local reflex control strategy using PAM pressures, we identified certain limitations: (1) Pressure changes in distal muscles proved insufficiently sensitive to detect upper-body perturbations. (2) Unsynchronized local feedback might cause individual leg responses to interfere with one another.

Considering these limitations and the role of load-based feedback in human motor control Duysens et al. (2000), Bastiaanse et al. (2000), GRF provides a more suitable global signal that captures the overall interaction between the body and the environment. Utilizing GRF to control PAMs can be interpreted as a form of FMC (force modulate compliance) (Sharbafi and Seyfarth, 2015). The stability of this force-modulated approach is theoretically supported by the Virtual Pivot Point (VPP) concept (Maus et al., 2010). Experiments show that the biological system inherently redirects the GRF vector to intersect at a VPP located above the CoM. This shifts the perturbed system behaviors from an unstable inverted pendulum into a marginally stable virtual pendulum. Consequently, small perturbations automatically generate appropriate restoring torques without requiring absolute posture feedback. Previous studies have demonstrated (Sharbafi et al. 2013a; Sharbafi et al., 2013b); Sharbafi and Seyfarth, (2015) that utilizing GRF feedback to modulate joint compliance can approximate VPP and therefore transfer the system into a pendulum model with stabilizable dynamics. Subsequent studies further exhibit the effectiveness of GRF-based control during dynamic locomotion (Sarmadi et al., 2018; Koseki et al., 2025). Since a PAM’s compliance is an intrinsic, pressure-dependent property, active pressure regulation enables continuous modulation of actuator compliance. In this study, we focus on the horizontal component of the GRF, $F_{x}\left(t\right)$ , which better reflects the rotational perturbation from CMG than the vertical component.

The high-level reflex is governed by a threshold-triggered logic. To ensure system stability, the reflex is activated only when the filtered horizontal force $\left|F_{x}\left(t\right)\right|$ first exceeds the predefined threshold $F_{th}$ (at $t_{\mathrm{trig}}$ ). Once triggered, the pressure modulation remains active until the force signal completes its first primary oscillation and returns to zero (at $t_{\mathrm{zero}}$ ). After returning the force direction, the activation will be set to zero to avoid unwanted oscillations. The reflex pressure component, $p_{m}^{r}\left(t\right)$ , is defined in Equation 1 as

$$p_{m}^{r}\left(t\right)=\left\{\begin{array}{cc} k_{m}\cdot |F_{x}\left(t\right)| & \text{if }t_{\mathrm{trig}}\leq t<t_{\mathrm{zero}}\\ 0 & \text{otherwise} \end{array}\right.$$ (1)

where $k_{m}$ is muscle-specific gains. The triggering time $t_{\mathrm{trig}}$ and the mute-start time $t_{\mathrm{zero}}$ are defined by Equations 2, 3, respectively:

$$t_{\mathrm{trig}}=\min\left\{t||F_{x}\left(t\right)|\geq F_{th}\right\}$$ (2)

$$t_{\mathrm{zero}}=\min\left\{t>t_{\mathrm{trig}}|F_{x}\left(t\right)\cdot F_{x}\left(t_{\mathrm{trig}}\right)\leq 0\right\}$$ (3)

The total desired pressure for each PAM, denoted as stimulation signal STI(t) , is defined according to Equation 4:

$$STI_{m}\left(t\right)=\text{sat}\left(p_{m}^{d}\left(t\right)+p_{m}^{r}\left(t\right)\right)$$ (4)

where $p_{m}^{d}\left(t\right)$ represents the initial pressure, and $p_{m}^{r}\left(t\right)$ is the dynamic reflex component. We saturated the pressures up to 0.6 MPa for safety reasons.

To minimize mechanical wear of the solenoid valves, the low-level pressure regulation utilizes a PD control law with an integrated deadzone to track the set-point $STI_{m}\left(t\right)$ . The error signal can be expressed in Equation 5 as:

$$e_{m}\left(t\right)=STI_{m}\left(t\right)-p_{m}^{m}\left(t\right)$$ (5)

where $p_{m}^{m}\left(t\right)$ is the measured pressure from sensors. The control output $u_{m}\left(t\right)$ can be formed as shown in Equation 6:

$$u_{m}\left(t\right)=\left\{\begin{array}{cc} \text{inject} & \text{if PD}\left(e_{m}\right)>\delta \\ \text{exhaust} & \text{if PD}\left(e_{m}\right)<-\delta \\ \text{hold} & \text{if }|\text{PD}\left(e_{m}\right)|\leq \delta \end{array}\right.$$ (6)

where δ represents the operation deadzone, PD is the proportional and derivative controller with $K_{p}$ and $K_{d}$ as gains, calculated in Equation 7:

$$\text{PD}\left(e_{m}\right)=K_{p}e_{m}+K_{d}{\dot{e}}_{m}$$ (7)

All control parameters used in this study are detailed in Table 1.

**TABLE 1:** Reflex parameters for active balance recovery experiments.

Reflex gain	Proximal (HAM, RF, GLU, IL)	Intermediate (POP, VAS)	Distal (GAS, SOL, TIB)	Perturb Level
Single $k_{m}$	0.04 MPa/N	0.08 MPa/N	0.10 MPa/N	3 Nm
Multiple $k_{m}$	0.03 MPa/N			5 Nm

Control Param	Description		Values	Unit
$F_{th}$	Reflex threshold		0.5	N
$P_{\mathrm{sat}}$	Desired pressure saturation		0.6	MPa
$K_{p}$	Proportional gain		0.6	–
$K_{d}$	Derivative gain		0.001	–
δ	PD control dead zone		0.025	–

### 2.3 Experimental setup

**FIGURE 3:**
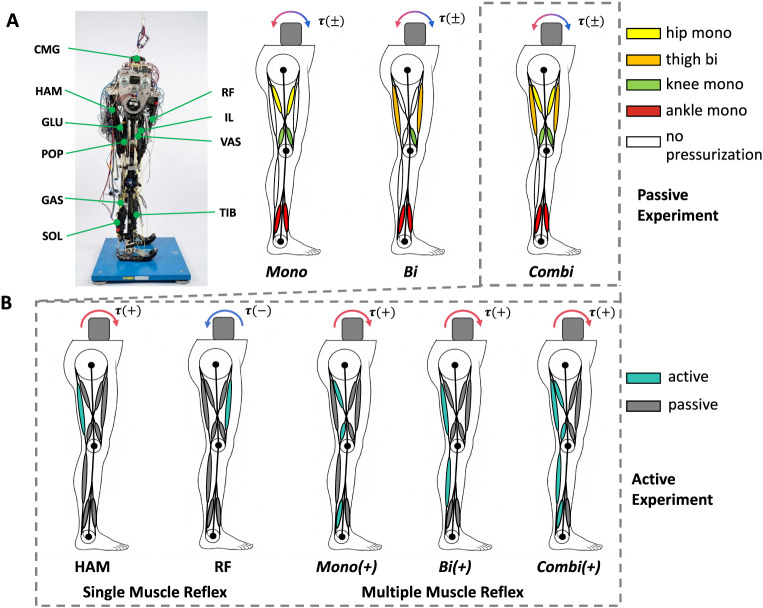
Experimental setup for perturbed standing recovery. **(A)** EPA-Walker with passive experiments: Three muscle configurations (*Mono*, *Bi*, and *Combi*) were evaluated to identify the morphological stability limits of different PAM arrangements. **(B)** Active Experiments: Implementation of GRF feedback controller on specific muscle sets to investigate reflexive balance recovery.

To understand the contributions of morphological properties and control mechanisms, as shown in Figure 3, this study is divided into passive (top) and active (bottom) perturbed standing recovery experiments.

#### 2.3.1 Stiffness optimization in simulation

Before doing the experiments, we ran simulations and optimizations to have reasonable pressure settings for the robot experiments. In the passive standing, we focused on three muscle morphological sets categorized by the joints each muscle spans: *Mono* (monoarticular PAMs only), *Bi* (predominantly biarticular PAMs), and *Combi* (hybrid of both), as color-coded in Figure 3A. GAS was excluded from either *Bi* or *Combi*. In *Bi*, the absence of an antagonistic pair for the GAS prevents the regulation of backward leaning. In *Combi* with preliminary robot tests, the current PAM configurations placed the Center of Pressure (CoP) around the forefoot. Adding GAS generates a plantarflexion force that shifts the CoP toward the curved toe, inducing instability. The exclusion of GAS has biological supports, where human studies Héroux et al. (2014) show that while the soleus remains tonic, the lateral gastrocnemius is largely inactive and the medial gastrocnemius is only intermittently active during standing balance.

The optimization was conducted in MATLAB/Simulink, utilizing a four-segment model with linear springs as substitutes for PAMs. To reduce the dimensionality of the optimization space and focus on the influence of hip–ankle stiffness distribution on standing stability, we assumed fixed knee stiffness and symmetrical stiffness in antagonistic muscle pairs. Such assumptions are also well compatible with the approximately symmetric configuration of the EPA-Walker platform. Consequently, the stiffness ratios for the hip monoarticular $\left(r_{hm}\right)$ , thigh biarticular $\left(r_{hb}\right)$ , and ankle monoarticular $\left(r_{am}\right)$ with respect to knee stiffness were defined as the input variables. To minimize standing energy and maximize stability, the cost function J is defined as:

$$J=c-\tau_{\mathrm{range}}+\lambda \cdot \|r{\|}^{2}$$ (8)

where $\tau_{\mathrm{range}}$ represents the tolerable perturbation torque range, c is a positive constant introduced to keep J non-negative without affecting optimization, set to 6 based on the scanned perturbation range. $\|r{\|}^{2}$ denotes the square norm of the stiffness ratios. For *Mono*, Bi, and Combi, r is equal to $\left(r_{hm},r_{am}\right),\left(r_{hb},r_{am}\right),\left(r_{hm},r_{am},r_{hb}\right)$ , respectively. λ=0.3 is a penalty term to avoid being too stiff, which is selected based on the relative range of $\tau_{\mathrm{range}}$ and $\|r{\|}^{2}$ . Empirically, within certain ranges (0.1–0.5), the selection of λ will not significantly shift the resulting ratios. The detailed setup for these simulations is provided in Supplementary Appendix 2. Specifically, VAS was incorporated into *Bi* to maintain an extended knee and avoid kinematic singularities inherent in purely biarticular configurations.

#### 2.3.2 Passive standing experiments

The optimal stiffness values found in simulations were mapped to PAM pressures for the experiments based on the PAM model from Mohseni et al. (2020). For each group, we conducted both forward and backward perturbations, with 10 repetitions in each direction, and recorded the number of successful recoveries. The evaluation metric for passive trials is the maximum tolerable perturbation. We identified this threshold by initiating relatively high CMG-generated perturbations (1 Nm) and monotonically decreasing the torque by 0.05 Nm until a success rate of 80% (8 out of 10 trials) was achieved.

#### 2.3.3 Active experiment: GRF-based reflex control

The active experiments are conducted based on *Combi*, which demonstrated superior robustness during the passive phase. In Figure 3B, we have single and multiple muscle reflex tests. We selected the muscles to be activated according to the perturbation direction. In forward perturbations, which tend to pitch the body forward, we targeted muscles in the back of the leg, including HAM, GLU, POP, SOL, and GAS, as they can generate the torque required for perturbation compensation. For backward perturbations, we selected the muscles on the front side of the leg, such as RF, IL, VAS, and TIB, that can counteract the CMG torque effects. For single muscle activations, only one muscle is activated by reflex; in multiple muscle activations, feedback was applied to all monoarticular muscles, all biarticular muscles, or the entire muscle set simultaneously. Based on the heuristic search, we found that 3 Nm is a tolerable perturbation value for a moderate number of active configurations. This is comparable to (but lower than) the exerted perturbation in the human experiment of Schumacher et al. (2019), considering the ratio of mass and inertia of humans to those of the robot.

We utilized two metrics for balance evaluation: the number of successful recovery trials and the mean standing time. The standing time is defined as the interval between the start of the perturbation and the time of falling (identified when the vertical ground reaction force ${\text{GRF}}_{z}$ drops below 95% of its initial static value). For a successfully balanced trial which does not fall, we set this time equal to 5 s . For the multiple muscle reflexes that are more capable than single muscle reflexes, we consider ±5 Nm perturbation, which is more comparable to a human perturbation recovery experiment.

Each setting was evaluated with seven repetitions for single-muscle experiments and five repetitions for multiple-muscle experiments. The number of trials was selected based on the practical constraints of hardware-intensive pneumatic robot experiments (Masuda et al., 2020; Matsumoto et al., 2023) and the need to ensure sufficient statistical sensitivity under the expected large effect sizes. This number of repetitions is capable of identifying dominant morphology–control effects, which are further supported by physical response patterns. Due to the ceiling effect caused by the 5-s truncation of stable trials, statistical significance of standing time was assessed using the nonparametric Kruskal–Wallis test followed by Dunn’s post-hoc comparisons between different reflex groups.

Table 1 summarizes parameters for different experiment groups. The control gains for single muscle groups were determined through a systematic parametric sweep within a range of 0.02–0.20. The sensitivity to gain variation was found to depend on the muscle group. Muscles with a strong contribution to balance recovery (e.g., HAM, RF, GAS, and SOL) exhibited a moderate stable operating range (±0.02) around the selected optimal gains, while larger gains led to over-compensatory behavior. In contrast, muscles with weaker contributions (e.g., GLU, IL, and POP) showed lower sensitivity to gain variations, but generally failed to achieve effective stabilization regardless of the exact gain choice. In multiple muscle configurations, gains were kept identical across all contributing PAMs and were set lower than those in single muscle trials. This reduction was necessary to prevent control instability and over-compensation, as the collective stiffness of multiple active pathways could otherwise lead to unwanted excessive joint displacements.

## 3 Results

### 3.1 Passive balancing

**FIGURE 4:**
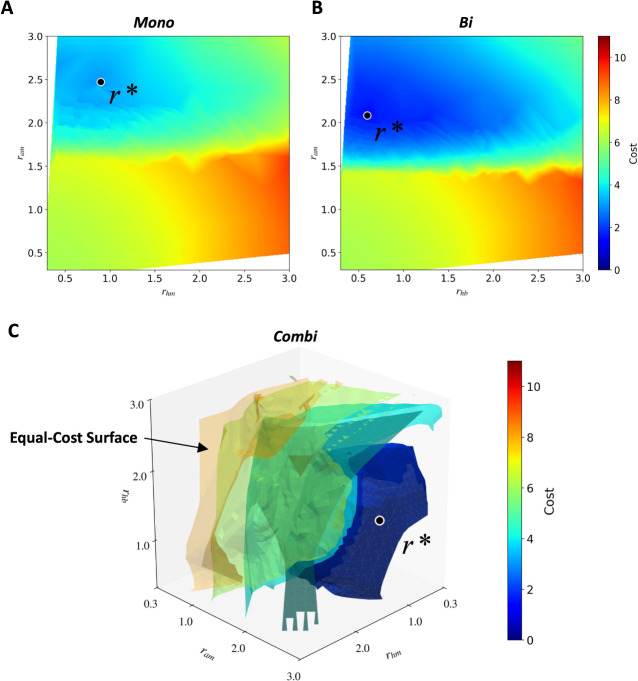
Simulation-based optimization of muscle stiffness effects in robustness. The cost function (including the tolerable perturbation torque range and a penalty factor for muscle stiffness, defined in (8)) variations with respect to different muscle stiffness ratios are shown for **(A)** *Mono*, **(B)** *Bi*, and **(C)** *Combi*. Each axis represents the stiffness ratio of the hip monoarticular $\left(r_{hm}\right)$ , thigh biarticular $\left(r_{hb}\right)$ , and ankle monoarticular $\left(r_{am}\right)$ relative to the knee. The color gradient represents the cost function value, with the optimal configuration for each group marked by r* . The transparent surface in **(C)** denotes contours of equal cost values.

The optimization results are illustrated in Figure 4. The colorbar represents costs defined in Equation 8. The blue areas represent low-cost zones and suggest greater robustness against perturbations. For *Mono* (Figure 4A), $\left(r_{hm},r_{am}\right)$ were optimized, with a global minimum at $r^{*}=\left(0.89,2.47\right)$ with almost 3 times higher ankle stiffness than hip stiffness. Stiffening the hip and softening the ankle yields a greenish color, showing lower robustness.

In *Bi* (Figure 4B), the minimum cost is $r*=\left(r_{hb},r_{am}\right)=\left(0.59,2.08\right)$ , which requires about 3.5 times higher stiffness at the ankle joint compared to the biarticular hip joints. In general, increasing biarticular PAM stiffness to about 2 while keeping ankle with a comparable stiffness found in r* does not significantly change the robustness, while lowering ankle stiffness diminishes it considerably.

For *Combi* (Figure 4C), a sequence of surfaces with equal costs were depicted, and the lowest cost r* is encapsulated at $\left(r_{hm},r_{am},r_{hb}\right)=\left(0.67,2.42,0.78\right)$ . It shows a similar trend of needing high stiffness at the ankle joint, while there is room to tune mono- and bi-articular hip muscles without significantly losing robustness.

Considering the optimal ratio (approximately proximal-to-distal is 1:3) and PAM model from Mohseni et al. (2020), we assigned proximal muscle pressures to 0.2 MPa and ankle muscles to 0.4 MPa . Due to mechanical constraints that prevent knee hyperextension, we set VAS pressures to 0.3 MPa and reduce POP to 0.1 MPa to ensure upright posture. The specific pressurization levels are summarized in Table 2.

**TABLE 2:** Passive standing pressure setting based on optimization results.

Parameters		HAM	RF	GLU	IL	POP	VAS	GAS	SOL	TIB
Passive pressure $P_{0}$ (MPa)	*Mono*	–	–	0.2	0.2	0.1	0.3	–	0.4	0.4
	*Bi*	0.2	0.2	–	–	–	0.3	–	0.4	0.4
	*Combi*	0.2	0.2	0.2	0.2	0.1	0.3	–	0.4	0.4

**FIGURE 5:**
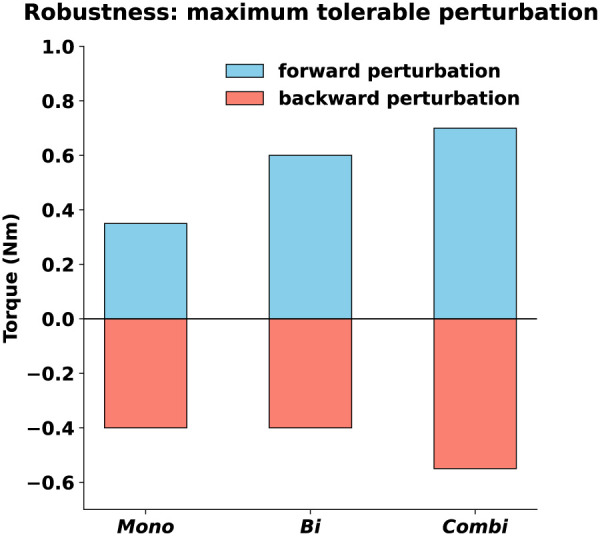
Robustness comparison using maximum tolerable perturbation across different groups in passive standing. The robustness was recorded based on the balance success rate (above 80%) from ten repeated trials.

The maximum tolerable perturbation for passive standing across different experimental groups is shown in Figure 5, with blue bars denoting forward perturbations and red bars representing backward perturbations. The results demonstrate that the *Combi*, which integrates both mono- and bi-articular muscles, achieves the highest robustness against perturbation, withstanding a torque range from −0.55 Nm to +0.70 Nm . In comparison, the *Bi* (utilizing biarticular HAM and RF muscles) outperforms the *Mono* (relying on GLU and IL) in forward disturbance rejection, sustaining up to +0.60 Nm compared to only +0.35 Nm for the latter. On the other hand, both *Mono* and *Bi* show similar performance in backward perturbations, each resisting up to −0.40 Nm .

### 3.2 Active balancing

#### 3.2.1 Reflex on single muscles

**FIGURE 6:**
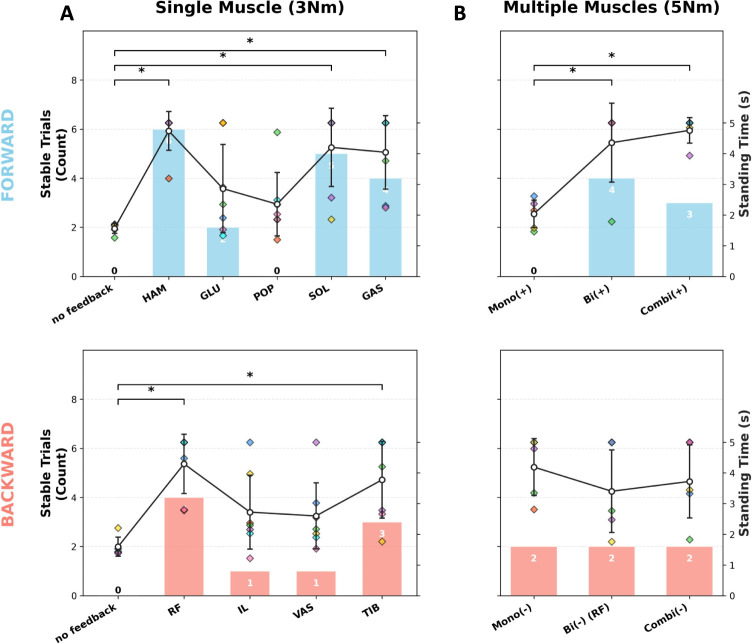
Quantitative evaluation of active balance recovery performance across different muscle configurations. **(A)** Performance of single-muscle reflex strategies under ±3 Nm perturbations; **(B)** Performance of multiple muscle groups under ±5 Nm perturbations. Bar charts indicate the number of stable trials, while scatter points and error bars represent the mean and distribution of standing times for all trials. Statistical significance is indicated by * (p<0.05) , based on the Kruskal–Wallis test followed by Dunn’s post-hoc tests.

A comparison of different PAMs’ activations besides no feedback as a reference group is depicted in Figure 6A, where colored bars represent the number of stable trials and black solid lines indicate mean standing time. In forward perturbations, HAM, GAS, and SOL are the effective muscles for maintaining balance.At the 3 Nm level, HAM achieved the highest success rate (6/7) and mean standing time (4.74 s) , exhibiting a significant improvement over the no-feedback group (p=0.0023) . SOL and GAS also significantly improved robustness. SOL achieved 5/7 successful trials with a mean standing time of 4.20 s (p=0.0155) , while GAS achieved 4/7 successful trials with a mean standing time of 4.04 s (p=0.0336) . In backward perturbations, RF was the most responsive muscle, stabilizing the robot in 4/7 trials and significantly extending the standing time compared to the no-feedback group (p=0.0036) . TIB also showed significance (p=0.0360) , although the stable counts are less than half of the total trials. Other muscles are less effective as the robot failed to recover in most of the trials. Overall, the robot demonstrated better balance recovery in the forward direction compared to the backward direction.

**FIGURE 7:**
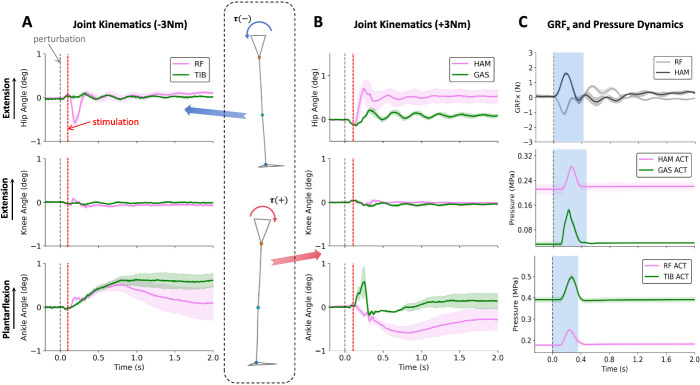
Representative kinematics and dynamics responses in single muscle reflex experiments. **(A,B)** Joint angle trajectories for hip, knee, and ankle under ±3 Nm perturbations using single muscle reflexes: RF and TIB in **(A)**, and HAM and GAS in **(B)**. Vertical dashed lines indicate the timing of perturbations (gray) and subsequent stimulation (red). **(C)** Horizontal GRF comparing HAM and RF reflex, and the representative muscle pressure responses. The hip muscle (HAM and RF) pressures are plotted in violet lines, while ankle muscle (GAS and TIB) pressures are denoted in green lines. The blue shaded areas denote CMG perturbation durations.

Figure 7 shows the kinematics and dynamics response (stable trials) of the perturbation recovery. Subfigures A,B exhibit distinct kinematics behaviors of using hip (HAM, RF, denoted as violet lines) and ankle (GAS, TIB, denoted as green lines) muscles for recovery in the backward and forward direction, respectively. We chose these four representative muscles as they perform better compared to other cases. The activation of HAM and RF primarily rectifies proximal joint orientations, whereas GAS feedback predominantly regulates distal joint displacements. TIB is much less effective, where no significant corrective displacements can be observed. In C, the perturbation-reflex GRF patterns of thigh biarticular muscles and four muscles measured pressures are demonstrated, in which the oscillation of horizontal GRF can be effectively damped out due to the GRF-based reflex control in the corresponding muscle.

#### 3.2.2 Reflex on multiple muscles

To further improve robustness to recover from larger perturbations (5 Nm) and to understand muscle interactions, we implemented reflex strategies targeting a combination of different muscles: monoarticular, biarticular, and combined, as pre-defined in passive experiments. The results are shown in Figure 6B. In forward perturbation, there are significant balance differences between the groups. The *Bi(+)* and *Combi(+)* configurations achieved high stability, both significantly extending standing time compared to the *Mono(+)* group (p=0.0411) . *Bi(+)* reached an 80% success rate and *Combi(+)* exhibiting the longest standing time. In contrast, the *Mono(+)* group failed in all trials. During backward perturbations, however, the influence of muscle interaction was less dominant (p=0.6321) , with *Mono(−)*, *Bi(−)*, and *Combi(−)* each achieving two successful recoveries.

**FIGURE 8:**
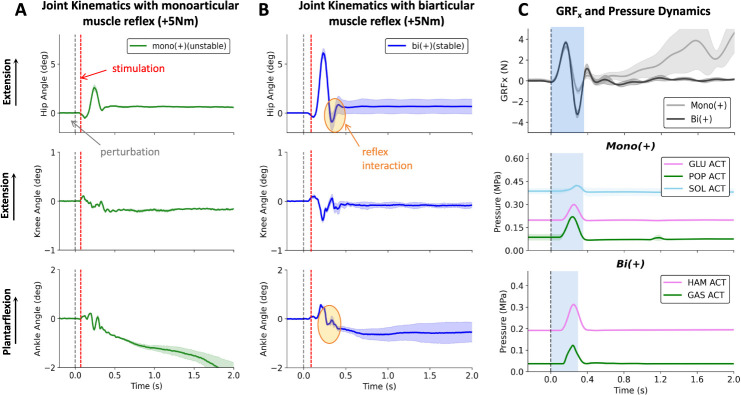
Representative kinematics and dynamics responses in multiple muscle reflexes experiments. **(A)** *Mono(+)* using GLU, POP, and SOL under +5 Nm perturbations. **(B)** *Bi(+)* using HAM and GAS under +5 Nm perturbations. Orange circles highlight multiple muscle reflex interactions. Vertical dashed lines indicate the timing of perturbations (gray) and subsequent stimulation (red). **(C)** Horizontal GRF and muscle pressure changes. The blue shaded areas denote perturbation durations.

Figure 8A,B, illustrates the joint angle excursion after forward perturbation for two configurations: *Mono(+)*, involving the activation of monoarticular muscles (GLU, POP, and SOL), and *Bi(+)*, in which the HAM and GAS biarticular muscles will be activated. In A, *Mono(+)* group shows isolated reflex responses and subsequent falls with dorsiflexion of the ankle joint. While in B with *Bi(+)* configuration, the joint trajectories at the hip and ankle exhibited significant peaks in reflex-recovery motions. These two angles also demonstrated coordinated effects at hip and ankle joints (shown by orange ovals) after the reaction of the muscles to perturbation. Figure 8C further highlights their distinct post-perturbation behaviors. Although the reflexive pressurizations of *Mono(+)* and *Bi(+)* are rather comparable, the horizontal GRF exhibits completely different patterns with the divergence of *Mono(+)* and convergence of *Bi(+)*.

## 4 Discussion

### 4.1 Passive standing

The robustness of the passive standing is primarily investigated through a simulation study of muscle morphological effects, specifically the stiffness of artificial muscles that can be applied later to the robot by tuning PAM pressures. Stiff ankle muscles (2.5–4 times stiffer than other muscles) are necessary to maintain postural balance with acceptable robustness against perturbations. In Figure 4, the robust regions (blue areas) are mainly located within higher $r_{am}$ , suggesting that insufficient ankle stiffness fails to provide the necessary torque to counteract the body’s gravitational load. These are in line with human experimental studies that reveal the significance of Soleus muscle force during quiet standing (Wickiewicz et al., 1983; Kouzaki and Fukunaga, 2008). However, it was shown that ankle muscle alone with the intrinsic mechanical stiffness cannot stabilize balance during quiet standing (Loram and Lakie, 2002; Morasso and Sanguineti, 2002; Vlutters et al., 2015). Interestingly, our simulations show that adding biarticular muscles rather than monoarticular muscles at the proximal joints not only increases robustness (darker blue in Figure 4B compared to Figure 4A), but also reduces the required stiffness ratio at the ankle joint from 2.47 to 2.08.

Passive standing experiments using PAM pressures, inspired by optimization results (see Table 2), confirmed the simulations by comparing *Bi* with *Mono*. The morphological functions of thigh biarticular muscles (HAM and RF) provide more significant effects on postural balance than monoarticular muscles (GLU, IL, and POP), particularly in the forward direction (+ 0.6 Nm versus + 0.35 Nm ), supporting previous findings about biarticular muscle roles in balance control (Sharbafi et al., 2016). Biarticular thigh muscles, configured with equal thigh and shank lengths and hip-to-knee lever arm ratio close to 2 (satisfied by EPA-Walker), produce corrective forces perpendicular to the leg axis Hof, (2001), Schumacher et al. (2020) with almost no cross-talk (axial force). These forces are essential for modulating body angular momentum during upper-body pitch recovery during locomotion tasks (Wells and Evans, 1987; Doorenbosch and van Ingen Schenau, 1995; Takahashi et al., 2023). Generating a similar perpendicular force with monoarticular muscles requires larger muscle forces at both the hip and knee joints, and the generated axial force must also be compensated. This could explain why biarticular thigh muscles can better support posture balance even in passive mode. The simulation results also validate this finding, as the optimal ratio r* in (Figure 4A) (0.89,2.47) is larger than that in B (0.59,2.08) .

Due to the asymmetric leg structure, including foot configuration (almost no lever arm behind the ankle joint), and to counteract backward falling, and knee mechanism avoiding hyperextension, the passive RF muscle cannot improve robustness against backward perturbations. However, the contribution of all muscles together in the configuration of *Combi* provides higher robustness compared to configurations concentrating on either mono- or bi-articular muscles, as shown in Figure 5. This means that morphological interaction can partially compensate for mechanical limitations.

### 4.2 Effects of muscle reflexes

Biomechanical studies revealed that activating muscles not only increases postural stability but also reduces measured ankle stiffness to below the critical stiffness, indicating a more efficient balance strategy (Loram and Lakie, 2002; Casadio et al., 2005). In alignment with the morphological functions shown in passive experiments, Figure 6 demonstrated that reflex controls on thigh biarticular muscles (HAM and RF) are most effective to increase robustness against perturbations. Similarly, in CMG-induced human perturbation experiments, Schumacher et al. (2019) identified that thigh biarticular muscles are dominant in the first response interval (100−150 ms) and are consistently involved in later intervals (≥170 ms) . GAS and SOL also exhibit sufficient capability of recovery as shown in Figure 6A, as they can provide ankle plantarflexion torque during body forward leaning and regulate the leg as an inverted pendulum to counteract upper-body rotational perturbation and balance the whole body. Such balance recovery strategies were also observed in other human perturbed standing studies (Horak and Nashner, 1986; Runge et al., 1999). The observed improvements associated with biarticular-related conditions, including HAM, RF, and the *Bi(+)* configuration, were consistently statistically significant compared with the no-feedback and monoarticular conditions, further supporting the dominant role of biarticular muscle function in perturbation recovery.

For moderate perturbations (3 Nm) , single-muscle reflexes, such as HAM, may provide sufficient capacity to maintain balance. As discussed above, biarticular thigh muscles showed superior performance in perturbation recovery compared with the other muscles considered. Their effectiveness appears to depend on two main factors: the magnitude of their mechanical effect and the speed of the sensorimotor loop. In terms of response speed, proximal muscles have an inherent advantage because, being closer to the perturbation source generated by the CMG, they can act more rapidly. In terms of effect magnitude, modulation of the GRF direction and the center-of-pressure position plays a central role. Biarticular thigh muscles can produce the fastest response while exerting the strongest influence on GRF direction without substantially changing GRF magnitude. This likely explains their high effectiveness in balance recovery, as reflected in both the success rate and the standing time. Although ankle muscles are located farther from the perturbation source, they can regulate the CoP, which may also play an important role in post-perturbation stabilization. Figures 7A,B supports these arguments by comparing the responses of a representative biarticular thigh muscle and an ankle muscle for both perturbation directions. The effects of RF and HAM at the hip joint can be seen to be both faster and larger than those of TIB and GAS. In addition, the implemented GRF-based feedback control may help compensate for the proximal-to-distal mechanical delay, as illustrated in Figure 7C. Therefore, although proximal and distal muscles are activated simultaneously in different experiments, their effects on the hip joint, which is closest to the perturbation source and plays a key role in rejecting the disturbance, differ substantially. This interpretation of muscle effectiveness in perturbation recovery may also help explain findings from several previous biomechanical studies (Horak and Nashner, 1986; Runge et al., 1999; Schumacher et al., 2019; Vlutters et al., 2015; Mohseni et al. 2024; Mohseni et al., 2025).

For larger perturbations (5 Nm) , robustness can be improved by the coordinated interaction of multiple muscles with suitably reduced gains to maintain stability. Consistent with this, human studies have reported synergistic activation of different muscles during balance recovery (Torres-Oviedo and Ting, 2007; Schumacher et al., 2020). In our analysis, *Bi(+)* achieved the best overall performance, with an 80% success rate (Figure 6B). Interestingly, although *Combi(+)* resulted in only three successful recoveries, its average standing time exceeded that of *Bi(+)*. This suggests that, even when the more complex system does not fully stabilize, it may still slow the onset of falling. Such behavior implies that further tuning of the *Combi(+)* control parameters could improve robustness. However, the results also indicate that incorporating additional muscles increases the complexity of reflex-control tuning, and without effective coordination, the extra contributions may even impair robustness. Overall, this highlights the trade-off between control complexity and task performance (Brockett, 1997).

While *Combi(+)* did not provide additional benefit in terms of robustness, we compared the kinematic behavior of the monoarticular and biarticular combinations in Figures 8A,B to better understand their different roles in perturbation recovery. A first observation is that *Mono(+)* shows smaller changes in hip angle than *Bi(+)*. However, this is not necessarily advantageous, because in *Mono(+)* the perturbation effect is transferred mainly to the ankle joint, which tends to destabilize it. Thus, the smaller hip deviation does not indicate better recovery if the whole-body posture cannot be stabilized. In contrast, the biarticular combination distributes the perturbation more effectively and promotes stability by regulating GRF direction and magnitude in a more balanced manner, as evidenced in Figure 8C. Given the comparable air injections in both configurations, the enhanced stability in Bi(+) highlights the inherent efficiency of biarticular morphology in translating GRF feedback signals into postural stability. In *Bi(+)*, the HAM + GAS combination effectively integrates the compensatory effects of the individual muscles, producing faster responses at both the ankle and hip joints (Figure 8B) than the isolated actuation of either HAM or GAS shown in Figure 7B. Hip joint displacement can be regulated closer to the initial posture, while the ankle joint enables a rapid corrective response followed by passive damping (Figure 8B, orange ovals). The coupling between HAM and GAS introduces a functional inter-joint interaction: HAM activation contributes to ankle dorsiflexion through its effect on shank motion, while GAS activation assists hip flexion through knee flexion. This coordinated coupling across joints substantially enhances recovery stability.

### 4.3 Actuation and control

A robotic platform for studying human-like neuromechanical control should reproduce key properties of the musculoskeletal system. Most existing bipedal robots, however, are based on rigid structures and electric motor actuation, which limits their suitability for investigating such principles. In contrast, this study employs EPA-Walker Silva et al. (2024), a soft robotic platform actuated by pneumatic artificial muscles (PAMs). A major advantage of PAMs is their biomimetic nature, as they approximate important mechanical characteristics of biological muscles (Daerden and Lefeber, 2002; Mohseni et al., 2020; Klute et al., 1999). This facilitates reverse engineering and the implementation of muscle-based motor control strategies. At the same time, PAM-based control is inherently challenging because pneumatic actuation is affected by valve nonlinearities, dead zones, and muscle hysteresis. In EPA-Walker, the solenoid on/off valves exhibit a substantial dead zone, typically causing static pressure errors of about 0.04 MPa , while the PAMs themselves show hysteresis Tondu (2012) caused by friction between fibers and tube. These factors contribute to the relatively high variance observed in the active control experiments. Even so, the implemented GRF-based reflex control was able to significantly enhance robustness compared with the passive configuration. In addition, repeated trials (ten in the passive condition and seven in the active configurations) helped reduce the impact of stochastic errors and support the statistical validity of the observed trends. The agreement with biomechanical findings from comparable human experiments Schumacher et al. (2019) further suggests that EPA-Walker captures sufficient features of human musculoskeletal and sensorimotor function to serve as a meaningful platform for the reverse engineering of human neuromuscular control.

The hybrid and nonlinear dynamics in bipedal robot movement pose significant challenges for controller design that match morphological design. This will be highlighted when there is an external source of instability like perturbation in our study. The key question that was addressed with a parsimonious solution of using the GRF for coordinating different players in leg control through tuning their compliance in a so called FMC (force modulated compliance) control. The functionality of this idea was approved in several locomotion scenarios, such as balance control Ito and Kawasaki, (2000), Sharbafi et al. (2016), human gait template-based modeling Sharbafi et al. (2013b); Sharbafi and Seyfarth, (2015), Firouzi et al. (2022), robot control (Mohseni et al., 2022; Oehlke et al. (2016), or assistive device control for locomotion (Davoodi et al., 2023; Naseri et al., 2020; Zhao et al., 2019). Inspired by these findings, this study investigated the usability of the FMC concept for perturbation recovery during standing for the first time via adjusting one muscle pressure (equivalently the compliance) based on GRF feedback. As shown in Figures 7, 8C, the horizontal GRF clearly reflects the perturbation effect, confirming that this signal can serve informative feedback signal. It is important to mention that this force feedback can be used as a universal feedback signal to activate different muscles, as an alternative to the local force feedback (Sarmadi et al., 2017; Geyer et al., 2003). We already tested whether the local feedback can be used by measuring the pressure of the same muscle, and our investigations showed that the pressure changes in different muscles are either nonsignificant or too much delayed to enable perturbation recovery (Due to the ineffectiveness of this local feedback, we did not present them in this paper). Instead, the GRF which represents the main interaction of the robot body with the environment (here the ground) plays the role of an indicator to activate muscles to provide perturbation recovery. Our FMC-based control results support the previous findings of human motor control (Dietz, 1998; Duysens et al., 2000; Dietz and Duysens, (2000) and open a new door for the robot control to increase robustness even in non steady state conditions.

The posture stability of human upper body balancing was analyzed using the virtual pendulum concept Maus et al. (2010), and was later implemented on different locomotion models through GRF-based FMC approach (Sharbafi et al., 2013a; Sharbafi and Seyfarth, 2015). FMC has been proven to be an effective approximation of VPP Sharbafi and Seyfarth (2015), by utilizing GRF to continuously modulate joint compliance, the controller inherently redirects the GRF vector to converge so that it points toward a VPP. This redirection fundamentally transforms the system’s behavior from an inverted pendulum into a stable, regular pendulum suspended at the VPP. During perturbed standing, the upper body typically acts as an unstable inverted pendulum, requiring appropriate control for stabilization. We found that if the controller is not matching the morphological considerations, e.g., in *Mono(+)*, the GRF direction diverges (shown in $GRF_{x}$ plots in Figure 8C). Our preliminary experimental observations (not shown here) suggested that VPP position exhibits a controllable trend when switching muscle actuation from distal to proximal groups, indicating different robustness levels against perturbation. However, more experiments and validations need to be done in future work.

Building on our previous extension of the FMC concept, in which joint compliance is coordinated through GRF as a central informative feedback signal, the framework of concerted control was introduced Sarmadi et al. (2019). Implementations of this concept in more complex, multi-degree-of-freedom models showed that GRF can serve as an effective coordinating signal, much like a conductor in an orchestra. Combined with locally embedded mechanical compliance, this signal provides sufficient sensory information not only for generating stable gait but also for improving robustness against perturbations, for example, during walking Koseki et al. (2026), In Press). Motivated by these findings, the present study extends the application of concerted control to practical PAM-driven balance recovery by enabling the simultaneous activation of multiple muscles. The EPA-Walker actuation system provides the physical basis for such an implementation. The results demonstrate that selecting an appropriate muscle group, particularly the biarticular muscles, can substantially improve robustness, for example, increasing the recoverable perturbation magnitude from 3 Nm to 5 Nm in forward perturbations. The reflex controller further showed robustness against pressure variability and postural redundancy introduced by the valves and actuators. Although the realized pressure responses were slightly delayed (Figures 7, 8C), their activation period remained well aligned with the perturbation interval, preventing depressurization from causing secondary disturbances. Even in the least effective cases, such as GLU and POP in Figure 6A, the mean standing time remained significantly longer than in the no-feedback reference condition, supporting the effectiveness of the approach. In the multi-muscle reflex cases, further improvements may be achievable through muscle-specific gain tuning, although at the cost of greater control effort and complexity. Importantly, in this implementation of concerted control, local feedback is realized mechanically through the compliant behavior of the PAMs, comparable to an impedance or physical PD controller, while the activation and tuning of this compliance are governed by the central GRF feedback. Thus, the individual muscles do not need to communicate directly; their coordination emerges through the shared conductor signal provided by the GRF.

### 4.4 Limitations and future steps

The present study also shows certain limitations for further improvements. First, the optimization relied on a simplified muscle model, assuming symmetrical antagonistic pressures and excluding GAS to reduce computational complexity. While this provided a tractable starting point, it may not fully represent the specialized morphological role of GAS. Future work involving individual muscle optimization could explicitly include GAS and may further enhance robustness. Secondly, the GRF-based controller was less effective in backward recoveries (Figure 6A). This reduced performance is closely linked to the morphological constraints of the robotic model, which already limit backward robustness in passive standing (Figure 5). Moreover, in humans, backward balance recovery prominently involves TIB and VAS Schumacher et al. (2019), especially in later response intervals (≥170 ms) . On EPA-Walker, however, such strategies are less applicable because the lack of toes and small, lightweight feet cause TIB pressurization to produce foot rotation (heel lift) rather than effective CoP modulation. Finally, although the present number of repetitions was sufficient to identify large morphology–control effects with high statistical sensitivity, the experiments were not designed to resolve subtle differences between closely related control configurations.

The morphology-control synergy in this study is not restricted to the EPA-Walker platform. From a morphological perspective, our findings regarding spring stiffness and biarticularity provide a design template for enhancing the intrinsic stability of legged robots. From a control standpoint, the GRF-based FMC approach is grounded in VPP, an underlying principle that can be generalized to diverse robot platforms. Therefore, future developments will focus on redesigning the foot, besides using the hybrid actuation to improve robustness, particularly against backward perturbations. In addition, building upon our previous sequential human studies on perturbed standing and perturbed walking (Mohseni et al., 2024; Mohseni et al., 2025), we aim to extend these balance strategies to robotic implementations and more dynamic locomotion scenarios. These include walking, stepping recovery, and locomotion over uneven terrain, in order to further evaluate the generalizability of the proposed framework across different locomotor conditions.

### 4.5 Conclusion

In this study, we systematically investigated how morphology and muscle-based control contribute to perturbed standing using the bio-inspired EPA-Walker platform. Our findings reveal that thigh biarticular muscles function as the primary morphological and functional drivers of robustness. In passive standing, appropriate distal stiffness remained essential, but adding proximal biarticular muscles increased forward robustness beyond monoarticular configurations. In active standing, GRF-based reflex control further highlighted the dominant role of biarticular muscles: HAM and RF were the most effective single-muscle responses for moderate perturbations, while the biarticular combination showed the best overall performance for larger perturbations. These results are consistent with previous human perturbation studies and strengthen the view that biarticular thigh muscles play a central role in balance recovery.

More broadly, this work shows the value of physically verifying biomechanical findings with a bio-inspired robotic platform whose morphology and actuation are designed to capture key features of the human musculoskeletal system. The results also emphasize that robust perturbation recovery does not depend on control alone, but on a control strategy that matches the underlying morphological design. In this context, the GRF-based FMC/concerted-control framework provided a parsimonious and effective way to coordinate compliant actuation and enhance robustness in a PAM-driven biped. Ultimately, these findings establish EPA-Walker not merely as a bipedal robot, but as a formal tool for the reverse engineering of neuromuscular principles, bridging the gap between biological theory and robust robotic implementation.

## Data Availability

The raw data supporting the conclusions of this article will be made available by the authors, without undue reservation.
