# Supplementary material for: Morphology and control roles in perturbed standing recovery: a robotic study
Source: Front Robot AI. 2026 Jul 1;13:1840011. doi: 10.3389/frobt.2026.1840011 (PMC13368573; doi:10.3389/frobt.2026.1840011)
Supplement: Supplementary file 1 [file Supplementaryfile1.pdf]

frontiers

Supplementary Material

1 MECHANICAL DESIGN AND CMG

Table S1. Mechanical Design Parameters of the EPA-Walker and PAM Specifications

**Parameter**	**Value**	**Unit**
Total Height	1.28	m
Total Weight (Except CMG)	13.36	kg
Thigh Length (Carbon Fiber Tube)	0.47	m
Shank Length (Carbon Fiber Tube)	0.41	m
CMG Mass	1.2	kg
CMG Dimensions	$101 \times 92 \times 117$	mm^3
PAM Contractile Part Lengths		
(Mono-articular)		
TIB	16	cm
SOL	17	cm
VAS	25	cm
POP	16	cm
IL	28	cm
GLU	30	cm
(Bi-articular)		
GAS	35	cm
RF	30	cm
HAM	35	cm

Table S1 summarizes the mechanical design parameters of EPA-Walker. The Control Moment Gyroscope (CMG) provides perturbation torques based on the principle of reactionless actuation, meaning it generates torque by internally exchanging angular momentum rather than reacting against the ground. The system consists of a flywheel with a high moment of inertia (I_s) spinning at a high velocity (Ω). By actively rotating the gimbal frame at a velocity of $\dot{\gamma}$, a gyroscopic torque τ_v is generated along the orthogonal axis to induce pitch perturbations, calculated as $\tau_v = I_s \Omega \dot{\gamma} \cos\gamma$. As a comparison, our robot's weight is only around one-fifth of an adult. The perturbation level in active standing experiments (± 3 and 5 Nm) can be considered as around ± 20 Nm perturbations (medium) in the human body.

2 SIMULATION OF PASSIVE STANDING OPTIMIZATION

The passive dynamics of the robot were simulated using a sagittal four-link model (representing the foot, shank, thigh, and trunk) within MATLAB Simscape. To model foot-ground interactions, a spatial contact force block was implemented with a stiffness of 10^7 N/m, damping of 10^3 Ns/m, and static and dynamic friction coefficients of 0.5 and 0.3, respectively. The simulation utilized a fixed-step ODE4 solver (2×10^{-4} s). The physical parameters of each link are detailed in Table S2. To approximate passive biological damping, joint damping coefficients were set to constant values: $c_{hip} = 5$, $c_{knee} = 1$, and $c_{ankle} = 0.5$ $Nm \cdot /rad$.

Table S2. Parameters of the four-link rigid body model.

Link	Length (m)	Mass (kg)	Inertia (kg·m^2)	Leverarm (cm)
Foot	0.16	0.19	0.002	r_A=8
Shank	0.41	0.47	0.038	r_K=7
Thigh	0.47	1.21	0.075	r_H=14
Trunk	0.23	4.81	0.084	

PAMs were modeled as linear springs. Assuming that antagonistic springs are pre-tensioned and have equal stiffness and moment arms (r), the joint rotation($\Delta\theta$) leads to length change $\pm\Delta\theta \cdot r$ in antagonists. The total joint torque $\boldsymbol{\tau} = [\tau_H, \tau_K, \tau_A]^T$ relates to the angular displacements $\Delta\boldsymbol{\theta}$ through a stiffness matrix $\mathbf{K}$. By superimposing the stiffness contributions from monoarticular (k_i) and biarticular (k_{i2j}) muscles, the linearized relationship $\boldsymbol{\tau} = -\mathbf{K}\Delta\boldsymbol{\theta}$ can be derived as:

$$\begin{bmatrix} \tau_H \\ \tau_K \\ \tau_A \end{bmatrix} = - \begin{bmatrix} 2k_H r_H^2 + 2k_{H2K} r_H^2 & 2k_{H2K} r_H r_K & 0 \\ 2k_{H2K} r_H r_K & 2k_K r_K^2 + 2k_{H2K} r_K^2 + k_{K2A} r_K^2 & k_{K2A} r_K r_A \\ 0 & k_{K2A} r_K r_A & 2k_A r_A^2 + k_{K2A} r_A^2 \end{bmatrix} \begin{bmatrix} \Delta\theta_H \\ \Delta\theta_K \\ \Delta\theta_A \end{bmatrix} \tag{S1}$$

In the optimization, the monoarticular knee stiffness was fixed at $k_K = 6000$ N/m. The optimization variables were the stiffness ratios of the remaining m muscles relative to k_K, denoted as r_{hm}, r_{am}, r_{hb}. The objective was to maximize torque range ($\tau_{range} = \tau_+ - \tau_-$, where τ_+ and τ_- are maximum positive and negative tolerable torques, respectively.) while minimizing the total system stiffness to ensure energy efficiency. For different groups, the target ratio vectors $||\mathbf{r}||$ are different. τ_{range} was identified by scanning perturbation from -3 Nm to 3 Nm (in *Combi* from -4 Nm to 4 Nm) to find the stable ranges. In MATLAB, we implemented a global optimization strategy that repeatedly initiates the SQP-based *fmincon* solver from 50 random starting points within defined bounds (from 0.3 to 3).
